# Hormones and Sex-Specific Transcription Factors Jointly Control Yolk Protein Synthesis in *Musca domestica*

**DOI:** 10.4061/2009/291236

**Published:** 2009-11-05

**Authors:** Christina Siegenthaler, Peter Maroy, Monika Hediger, Andreas Dübendorfer, Daniel Bopp

**Affiliations:** ^1^Zoological Institute, University of Zürich, Winterthurerstrasse 190, 8057 Zürich, Switzerland; ^2^Department of Genetics and Molecular Biology, University of Szeged, Közép fasor 52, 6724 Szeged, Hungary; ^3^Institute of Molecular Biology, University of Zürich, Winterthurerstrasse 190, 8057 Zürich, Switzerland

## Abstract

In the housefly *Musca domestica*, synthesis of yolk proteins (YPs) depends on the level of circulating ecdysteroid hormones. In female houseflies, the ecdysterone concentration in the hemolymph oscillates and, at high levels, is followed by expression of YP. In male houseflies, the ecdysterone titre is constantly low and no YP is produced. In some strains, which are mutant in key components of the sex-determining pathway, males express YP even though their ecdysterone titre is not significantly elevated. However, we find that these males express a substantial amount of the female variant of the *Musca doublesex* homologue, *Md-dsx*. The *dsx* gene is known to sex-specifically control transcription of *yp* genes in the fat body of *Drosophila melanogaster*. Our data suggest that *Md-dsx* also contributes to the regulation of YP expression in the housefly by modulating the responsiveness of YP-producing cells to hormonal stimuli.

## 1. Introduction

Yolk proteins (YPs) are the nutritional basis for the developing embryo in all oviparous animals. In insects, YP are synthesized in the female fat body, secreted into the hemolymph, and then taken up by the oocytes during maturation. In some insect species, such as *Drosophila melanogaster* and *Musca domestica*, YPs are also produced by the follicle cells surrounding the growing oocyte [1–3]. Three YP genes have been identified in *Musca*: *Mdyp1*, *Mdyp2*, and *Mdyp3*. However, MDYP1 and MDYP3 share the highest degree of amino acid similarity (82.5%), whereas MDYP2 reveals only 58.9% similarity to MDYP1 and 63.5% to MDYP3. Transcripts of all three *Mdyp* genes are expressed in the fat body and in the follicle cells surrounding the developing egg chamber in Musca females [4]. In Musca, ovarian development is synchronized, and after about five days of maturation, oocytes are laid in a batch. Adams [5] described ten oocyte stages: Stages 1–3 are the previtellogenic stages when the developing cysts have not yet taken up YP. Stages 4–8 are the vitellogenic stages, characterized by the uptake of YP-synthesized by the follicle cells and from the hemolymph. Stages 9-10, finally, are the postvitellogenic stages, when YP uptake has stopped, the nurse cells are degenerating, and the egg is ready to be laid.

In many insect species, YP synthesis is controlled by a sex-specific hormonal regimen, in particular, by juvenile hormone (JH) and ecdysteroid hormone [6]. A burst of ecdysteroids after a blood meal, for instance, triggers YP production in females of *Aëdes aegypti* [7]. Hormones play also an important role in controlling YP expression in *Musca domestica*. For instance, a decline of JH levels by removing its site of production in the corpora-allata corpora-cardiaca complex results in a loss of YP expression in females; when these females are again supplied with JH or methoprene, a JH analogue, YP production is resumed [8, 9]. While application of methoprene cannot stimulate YP synthesis in males [8, 10, 11], injection of 20-hydroxy-ecdysone (20E), the active isoform of ecdysone, induces YP synthesis in both males and females, even when JH production is eliminated by removing the corpora-allata complex [10]. Thus, it appears that JH is rather a permissible factor in the regulation of YP synthesis, but not a controlling agent. In *Aëdes aegypti*, fat body cells require being exposed to high JH levels before ecdysteroids can trigger vitellogenin synthesis [12]. In Musca, JH may serve a similar function to make fat body cells competent to respond to high ecdysteroid levels with the production of YP.

**Figure 1:**
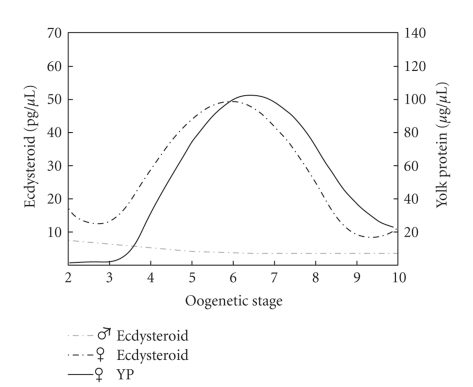
*Ecdysteroid and YP concentrations in hemolymph of Musca domestica*. In females, levels of ecdysteroids and yolk proteins oscillate in synchrony with the stages of ovarian development. Both levels reach a maximum in females with vitellogenic egg chambers. Stages 2-3: previtellogenic, stages 4–8: vitellogenic, stages 9-10: postvitellogenic stages. In males, the concentration of ecdysteroids is constantly low and no YP are produced. Summarized results from Adams and Filipi [13] and Adams et al. [14].

The main controlling agent of YP production in Musca appears to be ecdysone. In adult females, the ecdysterone level in the hemolymph is oscillating and followed by cyclic expression of YP, both reaching a maximum when synchronously developing oocytes arrive at stages 6-7 (Figure 1; Adams and Filipi [13], Adams et al. [14], Agui et al. [8]). Likewise, transcription of the *Mdyp* genes follows the same cyclic pattern: mRNAs of *Mdyp1* and *Mdyp3* are maximally abundant in the fat body and in the ovary at oocyte stages 4–8, and *Mdyp2* transcripts show a maximum at stages 5–9 [4]. Peak ecdysteroid levels in females are variable ranging between 18 pg/*μ*L [8] and 50 pg/*μ*L [14], depending on Musca strains used. In males, the ecdysteroid level remains continuously low at about 5 pg/*μ*L [8]. Injection of 20E into males induces transient YP expression [11]. Yet, ecdysteroids are not the only factor involved in YP regulation. In ovaryectomized females, ecdysteroids in the hemolymph drop to a very low, male-like level (<4 pg/*μ*L), but these females nevertheless continue to produce YP, if only at a low rate [8]. Furthermore, both allatectomized females and males start to produce YP after injection of 20E, but males are about 100 times less sensitive, and the response is significantly delayed [10].

**Figure 2:**
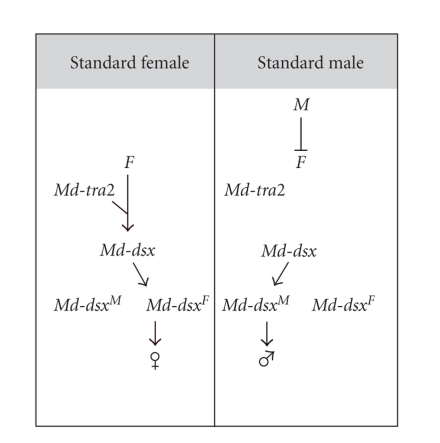
*Sex determination in Musca domestica*. Presence or absence of the male determinant *M* determines the sexual fate of the zygote. When *M* is present, it represses *F*, the female-determining switch gene. By default, *Md-dsx*, the *Musca* homologue of *doublesex*, expresses a male product *Md-dsx^M^* and male development follows [15]. When *M* is absent, zygotic *F* is activated. This activation depends on maternally supplied *F* activity [16]. Zygotically activated *F*, together with its cofactor *Md-tra2*, imposes a change in the splicing pattern of *Md-dsx*, which results in the production of the female variant *Md-dsx^F^* and female development [15].

Thus, it seems that in females additional factors are present which render the *Mdyp* genes more susceptive to activation by ecdysteroids. In this report, we demonstrate that *Md*-*dsx* is a likely candidate. In Drosophila, *dsx* is known to directly control the transcription of the three *yp* genes in fat body cells. The male variant, DSX^*M*^, represses YP transcription, whereas the female variant, DSX^*F*^, acts as an enhancer of YP transcription (reviewed in Bownes [17]). The *Musca* homologue, *Md*-*dsx*, produces sex-specific splice variants, Md-DSX^*F*^ and Md-DSX^*M*^ which structurally and functionally correspond to DSX^*F*^ and DSX^*M*^ in Drosophila [15]. As in Drosophila, the *Md-dsx* gene is proposed to be the terminal regulator in the sex-determining cascade, relaying the primary sex-determining signal to the sex-differentiating genes [15]. The primary signal in *Musca* sex determination is the male-determining factor *M* (for a review, see Dübendorfer et al. [18]). When *M* is present in the zygote, it represses the female-determining key gene *F* and, as a result, *Md-dsx* is spliced by default in the male mode producing transcripts, which encode Md-DSX^*M*^. When *M* is absent in the zygote, the zygotic *F* gene is activated by maternally supplied *F* activity [16] and, together with its cofactor *Md-tra2* [19], promotes the female splice mode of its downstream target *Md-dsx* to give rise to the female splice variant which encodes Md-DSX^*F*^ (Figure 2; Hediger et al. [15]). In natural populations, *M* factors are located at different sites in the genome, most commonly on the Y chromosome but also on any of the five autosomes or even on the X chromosome [20]. These *M* factors seem to differ in their capacity to repress the female pathway. For instance, *M* ^I^ (*M* on chromosome I) allows production of YP, a typically female-specific physiological trait, in otherwise normal and fertile males [21, 22]. Likewise, YP production is found in males with a female genotype. Such males arise due to homozygosity of a recessive mutant allele of the female determiner *F*, *F* ^man^ (Figure 2). Based on this finding, Schmidt et al. [21] suggested that these males express some residual *F* activity and concluded that *F* ^man^ is not a null allele, but rather a strong hypomorph.

In this study, we demonstrate that *M* ^I^ and *F* ^man^ males which express YP even when ecdysteroid hormones levels are low produce substantial levels of Md-DSX^*F*^ suggesting that *Md-dsx* contributes to the sex-specific regulation of *yp* genes in Musca.

## 2. Materials and Methods

### 2.1. Fly Strains

As control strains we used a wild-type, a *white* (*w*/*w*), and a multiply marked strain (*ac*/*ac*; *ar*/*ar*; *bwb*/*bwb*; *ocra*/*ocra*). These strains have standard-type sex determination: XY males and XX females. Autosomal markers used in this study are as follows:

 Chromosome I: *ac*—*ali curve*, curved wings,
 Chromosome II: *ar*—*aristapedia, *
 Chromosome III: *bwb*—brown body, *w*—white eyes,
 Chromosome IV: *Ba*—*Bald abdomen, *
 Chromosome V: *ocra*—ochre eyes,
 
*M* ^I^ strain: Male genotype XX; *M* ^I^ +/+ *ac*; *ar*/*ar*; *bwb*/*bwb*; *ocra*/*ocra*. Female genotype XX, *ac*/*ac*; *ar*/*ar*; *bwb*/*bwb*; *ocra*/*ocra*,
 
*F* ^man^ strain: Male genotype: XX; *ac*/*ac*; *F* ^man^/*F* ^man^. Female genotype: XX; *ac*/*ac*; *F* ^man^ *Ba* ^+^/*F* ^+^ *Ba*.

The strains were reared as described previously [21, 22].

### 2.2. SDS-PAGE and Western Blotting

Hemolymph samples of individual flies were collected by inserting a glass capillary into the ventral thorax. The samples were transferred into 13 *μ*L of 2x SDS sample buffered on ice, boiled for 5 minutes, and stored at −78°C. Oocyte stages in females were determined after taking the hemolymph samples, using the definitions of Adams [5, see introduction]. Preparations of ovaries and fat body extracts: flies were dissected in Musca Ringer's solution (7.5 g/L NaCl, 1 g/L KCl, 0.18 g/L CaCl_2_ *·*2H_2_O, 0.12 g/L NaHCO_3_, pH 7); ovaries or fat body were homogenized in 20 *μ*L 2x SDS, and insoluble material was removed by centrifugation. The supernatant was boiled for 5 minutes, and the samples were stored at −78°C. SDS-PAGE was carried out using the BioRad MiniProtean II System; entire samples (hemolymph) or 5 *μ*L (ovary and fat body samples) were loaded on a 12% SDS gel and separated by electrophoresis. Proteins were transferred to a 0.45 *μ*m nitrocellulose membrane (BioRad) in Tris-Glycin-Methanol. The membranes were incubated in blocking solution (4% low fat milk powder in TBS/0.05% Tween). The primary antibody (polyclonal anti-yolkprotein antibody, kindly provided by Dr. T. Adams) was diluted 1 : 20 000 in TBS/0.05% Tween with 1 mg/mL BSA; membranes were incubated in the antibody solution for 1 hour at room temperature. The antigen-antibody complex was detected using the alkaline phosphatase- (AP-) conjugated antirabbit antibody by Promega at a dilution of 1 : 7500 in TBST/1 mg/mL BSA.

### 2.3. Radio Immunoassays

Hemolymph samples were taken as described earlier and pooled on ice. The volume of the samples was measured using a micropipette. The pooled samples were dried in a SpeedVac for 45 minutes. The hemolymph samples were exhaustively extracted with 60 p.c. methanol. Aliquots of the extract were subjected to RIA. High avidity (20 000 fold) rabbit antiserum, raised against 20-hydroxyecdysone-6-ketoxime thyroglobulin conjugate, was used. RIA measurements were performed using a protocol described earlier [23], but with overnight incubations at 4°C. Results are expressed in 20-hydroxyecdysone equivalents, and normalised to hemolymph volume (*μ*L).

### 2.4. Northern Blot Analysis

Total RNA of 250 mg flies (~18 adult males or ~15 adult females) were extracted with the AGPC technique [24]. Poly(A)^+^ RNA was isolated using the Oligotex mRNA Maxi Kit (Qiagen). An amount of 1 *μ*g of mRNA per lane was fractioned by formaldehyde agarose gel electrophoresis, transferred to Hybond-N+ nylon membrane (Amersham) by blotting with 10x SSPE and cross-linked in a UV Stratalinker 2400 (Stratagene). Hybridizations were carried out in formamide hybridization solution at 42°C using 6*·*10^6^ cpm of labelled probe. Labelled antisense RNA probes were generated by in vitro transcription of PCR fragments of the *Mdyp* genes using T7 RNA polymerase (Promega) and *α*-^32^P-rCTP (Amersham).

### 2.5. Injection of 20E

In fact, 20-hydroxy-ecdysone (Sigma) was diluted in Musca Ringer's solution to concentrations of 10 ng/*μ*L and 1 *μ*g/*μ*L. Also, 1 *μ*L of these solutions was injected with a glass needle into the abdomen of 3d old males of the *M* ^I^ strain and of a standard strain as a control. Hemolymph of the injected flies was taken 24 hours after injection and analyzed by western blotting as described earlier.

### 2.6. RT-PCR

Poly(A)^+^ RNA was prepared as described earlier [15]. Also, 0.5 *μ*g mRNA was retro-transcribed using Enhanced AMV Reverse Transcriptase (Sigma), following the manufacturers protocols. Male and female transcripts of *Md-dsx* were amplified from cDNA by standard PCR techniques using Taq DNA polymerase (Promega). Primers used for *Md*-*dsx^F^* amplification: Primer C in the common exon 3, primer *F* in the female exon 4. Primers used for *Md*-*dsx^M^* amplification: primer C (common exon 3) and primer M in the male exon 5 (Figure 5). Samples of 5 *μ*L were taken after 24, 27, 30, and 39 PCR cycles and analyzed on a 1% agarose gel.

### 2.7. RNAi

The RNAi experiments of *Md-tra2* in Musca strain *M* ^I^ were performed by injections of dsRNA in early embryos as described in Burghardt et al. [19].

## 3. Results

### 3.1. Mdyp Expression in Males of the *M* ^I^ and *F* ^man^ Strains

**Figure 3:**
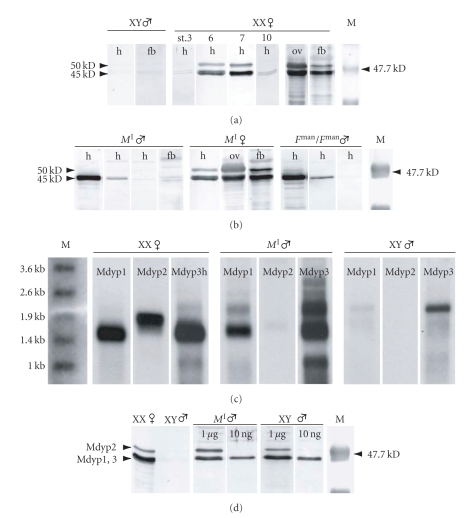
*RNA and protein expression of yp genes in M* ^I^ *and F* ^man^ *strains.* (a) Anti-YP crossreactivity in hemolymph (h), fat body (fb), and ovaries (ov) of males and females of a standard strain (st.: stages of ovarian development). The doublet at 45 kD represents the products of *Mdyp1* and *Mdyp3*, while the single band at 50 kD are products of the *Mdyp2* gene. (b) Anti-YP crossreactivity in males (left panel) and females (middle panel) of the *M* ^I^ strain; and in hemolymph of *F* ^man^ males (right panel). In each h lane the hemolymph of a single adult was loaded. Animals were 4d old when the samples were taken. (c) Northern blot of standard females (left panel), standard males (right panel) and *M* ^I^ males (middle panel) probed with labeled sequences specific for *Mdyp1*, *Mdyp2*, and *Mdyp3*. (d) Anti-YP crossreactivity in the hemolymph of single male after injection of 20E (1 *μ*g, 10 ng: amount of 20E injected), M: size marker.

We compared YP synthesis in males of the *M* ^I^ and *F* ^man^ strains with control males and females of a standard wildtype strain. Control males, regardless of their age, never show any traces of YP in their hemolymph or in fat body extracts when probed with a polyclonal anti-YP serum (Figure 3(a)). In extracts of control females, on the other hand, three cross-reacting bands are detected, one at 50 kD and a doublet at 45 kD. This pattern of YP-specific cross-reactivity is in accordance with data reported by Agui et al. [8] and Adams and Filipi [13]. The amount of detectable YP in the hemolymph depends on the stage of ovarian development. Low levels of the doublet are detected in females containing pre- or post-vitellogenic oocytes (stages 1–3 or 9-10), and their levels increase several folds during the vitellogenic stages reaching a maximum around stages 6 and 7 (Figure 3(a)). The 50 kD variant was only seen in females of this stage and its concentration was generally lower than that of the doublet proteins (Figure 3(a)). Agui et al. [10] and White and Bownes [4] observed that the amount of *Mdyp* transcripts in females also oscillates in synchrony with ovarian development. White and Bownes [4] reported that *Mdyp2*-mRNA was only detected in vitellogenic females suggesting that the 50 kD protein is a product of the *Mdyp2* gene. 

In the *M* ^I^ strain, about 80% of the males contain detectable levels of YP in the hemolymph. These males are otherwise morphologically normal and fertile. In the *F* ^man^ strain, the frequency of homozygous *F* ^man^ males producing YP varied between 4% and 40% in successive generations. We observed highly variable levels of YP expression in individual *M* ^I^ males even among siblings of the same age. The amount of YP in their hemolymph ranges from undetectable to levels comparable to that in vitellogenic females (Figure 3(b)). Variations in the level of circulating YP between individuals may in part be explained by gradual accumulation in the hemolymph, since males do not have an ovarian “sink” to dispose of secreted YP. A striking difference in the expression profile between males and females of the *M* ^I^ strain is the complete absence of the 50 kD variant in male hemolymph even when the concentration of the doublet reaches a level as high as in vitellogenic females. Similarly, the level of circulating YP in the hemolymph of *F* ^man^ males varied considerably from individual to individual and a 50 kD band was never detected even in extracts of the strongest YP-expressing individuals.

These findings suggested that the 50 kD band is absent in these males because the *Mdyp2* gene is transcriptionally inactive. Consistent with this conclusion we observed that, in *M* ^I^ males*, Mdyp2* transcripts are absent, in contrast to substantial amounts of *Mdyp1* and *Mdyp3 *transcripts present in these flies (Figure 3(c)). *Mdyp2* transcripts are only observed in vitellogenic females (Figure 3(c)). The presence of *Mdyp2* transcripts in female fat bodies excludes the possibility that *Mdyp2* is expressed only in ovarian tissues (Figure 3(a)). Absence of *Mdyp2* transcripts in *M* ^I^ and *F* ^man^ males can thus not be simply explained by the fact that these males have no ovaries.

Presence of the 50 kD variant, the putative product of *Mdyp2*, is limited to vitellogenic stages suggesting that *Mdyp2* expression can only be induced when the concentration of circulating ecdysteroid surpasses a high-level threshold. We tested whether injection of 20-hydroxy-ecdysone can trigger expression of the full repertoire of YP products in males if applied in high concentrations. Our results show that 10 ng of 20E was sufficient to induce expression of the 45 kD doublet in control males and in *M* ^I^ males, whereas a 100 fold higher dose, 1 *μ*g, was necessary to trigger the expression of the 50 kD band (Figure 3(d)). These results demonstrate that male tissues have the competence to produce MdYP2 and that transcriptional activation of *Mdyp2* requires higher concentrations of ecdysteroid hormones than activation of *Mdyp1* and *Mdyp3*.

### 3.2. Ecdysteroid Levels are not Increased in YP Synthesizing Males

Since YP synthesis in males can be triggered by injections of 20E, we examined whether expression of YP in *M* ^I^ and *F* ^man^ males results from elevated levels of ecdysteroid hormones. To this end we analyzed hemolymph samples of 3 days old flies. A radio-immuno assay (RIA) with an antiserum against 20E was conducted to measure the level of circulating hormones. Our results show that the ecdysteroid level in *M* ^I^ and *F* ^man^ males is low and comparable to that of control males (Table 1). The ecdysteroid levels measured in samples of control females and females of the *M* ^I^ and *F* ^man^ strains were 3-4 fold higher.

### 3.3. YP Synthesizing Males Express the Female Variant of Md-dsx

**Figure 4:**
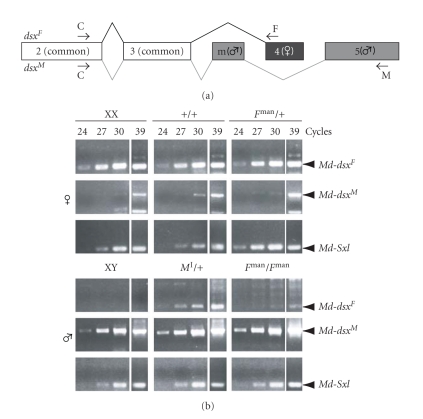
*RNA analysis of Md-dsx in M* ^I^ *and F* ^man^ *strains.* (a) Schematic representation of the *Md-dsx* gene and its splice variants. The male transcript consists of exons 2, 3, m, and 5; and the female transcript contains exons 2, 3, and 4 [15]. Arrows: primers used for amplification of the two *Mdsx* splice variants. C: common 5′ primer, F: female-specific 3′ primer, M: male-specific 3′ primer. (b) RT-PCR analysis of mRNA samples prepared from wildtype, *M* ^I^ and *F* ^man^ males and females. Of each amplification reaction, 5 *μ*L were removed after cycle 24, 27, 30, and 39 and analyzed on a gel to estimate transcript abundance. Level of *Md-Sxl* transcripts served as an internal control for mRNA quality and abundance.

Since ecdysteroid levels are not increased in *M* ^I^ and *F* ^man^ males, we concluded that other factors must be responsible for the induction of YP expression. The Musca *dsx* gene is a potential candidate in the light that YP genes in Drosophila are direct transcriptional targets of *dsx* [17]. In Musca, *Md-dsx* pre-mRNA is spliced differentially producing two sex-specific transcripts, *Md-dsx^M^* in males, and *Md-dsx^F^* in females (Figure 4(a); Hediger et al. [15]). We tested the possibility that *Md-dsx* is misregulated in *F* ^man^ and *M* ^I^ males.

We performed a semiquantitative RT-PCR to measure the levels of *Md-dsx^F^* and *Md-dsx^M^* transcripts in males and females of the *M* ^I^ and *F* ^man^ strain and of the standard XX/XY strain. As an internal standard, we monitored transcript levels of the homologue of *Sex-lethal (Md-Sxl)*, a gene that is expressed in males and females at equal levels [25]. Using sex-specific primer pairs (Figure 4(a)), our first experiment confirmed that standard XY males express abundant levels of *Md-dsx^M^* transcripts, but no *Md-dsx^F^*. On the other hand, *Md-dsx^F^* transcripts were readily detectable in control females (Figure 4(b)). Though transcripts of *Md-dsx^M^* were also recovered in females, their level was substantially lower than in males.

In males of the *M* ^I^ strain, we detected, in addition to* Md-dsx^M^* products, a significant amount of *Md*-*dsx^F^* transcripts (Figure 4(b)). Likewise, the *F* ^man^ males tested expressed clearly detectable amounts of *Md-dsx^F^* transcripts, though at a much lower level than in *M* ^I^ males. Thus, the presence of *Md*-*dsx^F^* in males correlates with YP expression. To test whether these genes are functionally interrelated we specifically silenced *Md-tra2*, a cofactor required for female-specific splicing of *Md-dsx*, by RNAi. Injection of *Md-tra2* dsRNA into early cleavage embryos has previously been shown to irreversibly shift *Md-dsx* splicing from the female to the male mode [19]. We observed an almost 30x reduction in numbers of *M* ^I^ males expressing YP when female splicing of *Md-dsx* was abolished by *Md-tra2* RNAi (Table 2). From this finding, it can be inferred that female expression of *Md-dsx* is required to activate *Mdyp1* and *Mdyp3* in a milieu low of ecdysteroid hormones. In line with this conclusion is the finding that transgenic expression of *Md-dsx^F^* is capable of inducing YP synthesis in standard males with a low ecdysteroid level (Table 2; Hediger et al. [15]). We thus conclude that *Md-dsx* is also involved in the control of this physiological response.

## 4. Discussion

In the housefly, *Musca domestica*, eggs develop synchronously after feeding [26]. Accordingly, YP production is coordinated with the onset of vitellogenesis and occurs in a cyclic fashion. In parallel to the YP concentration, ecdysteroid levels are oscillating in females, whereas, in males, they remain constantly low [13]. These results suggested that Musca relies on ecdysteroids for controlling YP synthesis in the fat body. However, studies based on exogenous application of ecdysteroids showed that male and female YP-producing cells respond differently [8]. In females, the activation threshold for YP synthesis appears to be significantly lower than in males. Since the fat body, the main site of YP synthesis, is present in both males and females, this difference cannot be attributed to tissue-specific regulation but is more likely to be an intrinsic feature imposed by the sex-determining cascade. A potential candidate that modulates the responsiveness to hormones in a sex-specific manner is the Musca *dsx* homologue, *Md-dsx*. In line with such a role, we find that the threshold of activating YP synthesis in males is substantially lowered when they express the female variant of *Md-dsx*. Since these males contain a normal low level of ecdysteroids in their hemolymph, we propose that binding of Md-DSX*^F^* to the promoter of *yp* genes enhances the binding of other stimulatory factors that are activated by ecdysteroids. The possibility that expression of *Mdyp1* and *Mdyp3* in these Md-DSX*^F^* expressing males is a consequence of a gain-of-function mutation in the *yp* genes themselves can be excluded for the following reasons: first, the *F* ^man^ and *M* ^I^ strains have different origins. Second, when *M* ^III^, a strong *M* factor on chromosome 3, is introduced in *M* ^I^ males, YP synthesis is completely abolished [22].

In Drosophila, sex-specific variants of *dsx* bind to the 125 bp Fat Body Enhancer (FBE) in the intergenic region of *yp1* and* yp2* genes to either enhance the basal transcription rate in females (DSX^*F*^) or to completely repress it in males (DSX^*M*^) [27–30]. Likewise, it has been shown in the lepidopteran species *Bombyx mori* that expression of the female-specific products of the *dsx* homologue is sufficient to induce vitellogenin synthesis in males [31]. Sequences that match the consensus *dsx* binding sites were found in the promoter region of the Musca yolk protein gene 1 (*Mdyp1*; Tortiglione and Bownes [32]). In addition, these authors showed that Drosophila DSX can bind to these putative binding sites in *Mdyp1* promoter in vitro. Although these upstream sequences of *Mdyp1*, when introduced into the genome of Drosophila, were able to drive expression of a *lacZ* reporter in a tissue-specific pattern identical to that of the endogenous *yp* genes, sex-specificity was not conferred [32]. Based on this finding, the authors suggested that *Mdyp* genes are not responsive to the action of sex-specific *dsx* variants and that such transcription factors thus may play no or only a minor role in controlling YP synthesis in the housefly. However, it can be argued that the promoter sequences tested did not contain all enhancer elements critical for conferring sex-specificity. Also, divergent evolution of *dsx* and the *yp* genes, since the separation of *Musca* and *Drosophila* is estimated 120 mya, may have prevented a functional interaction between the *Musca* yp promoter and the *Drosophila* transcriptional factors conferring sex-specificity. This apparent lack of sex-specific regulation closely resembles that of *yp3* in *Drosophila* [33]. It has been suggested that *yp3* regulation has diverged from that of *yp1* and *yp2* and may require additional hormonal inputs to achieve sex-specificity similar as in *Musca* and *Calliphora* [33].

**Figure 5:**
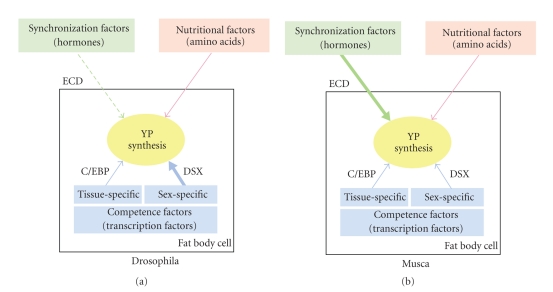
Model for YP regulation. Synthesis of YP in the fat body is regulated by a combination of different nonautonomous (green and red boxes) and autonomous (blue boxes) factors. The relative contribution of each depends on the mode of oogenic development. In fly species with continuous egg production, for example, Drosophila, competence factors play a more relevant role than synchronization factors. While in fly species with synchronized production of eggs, for example, Musca, hormones such as ecdysteroids (ECD), which control YP synthesis cyclically, have a more significant contribution than the autonomous sex-specific regulators.

Based on our results, we propose the following model for regulation of YP expression in *Musca domestica* (Figure 5). It is the combination of several different inputs that regulates the level of *yp* transcription in the fat body. A major contribution comes from the endocrine system to attune the production of YP not only with the synchronized maturation of oocytes but also with the availability of food resources. Both of these signals are nonautonomously transduced to the fat body cells. The ecdysteroid hormone seems to be the major component in reconciling the cyclic progress of vitellogenesis and availability of YP and therefore can be referred to as a synchronization factor. Also, hormones play a critical role in assessing the availability of nutrients and other environmental conditions relevant to egg production. In addition, YP synthesis depends on signals that confer sex- and tissue-specificity. These signals act cell-autonomously in the fat body cells and can be referred to as competence factors (Figure 5). In Musca, they play a minor but relevant role in modulating the responsiveness of *yp* genes to the nonautonomous signals. Presence of the female-specific variant of Md-DSX acts as a stimulatory factor, which upon binding to the *yp* promoters, in particular, that which drives expression of *Mdyp1* and *Mdyp3*, enhances transcription in the presence of ecdysteroids. The male-specific variant, on the other hand, may have the converse effect by inhibiting rather than stimulating hormone-induced transcription. The molecular nature of these interactions remains to be investigated. Apart from sex-specificity, a different set of transcription factors is needed to confer tissue-specificity. Some of these factors have been identified in Drosophila to be activators, such as the CCAAT/enhancer-binding protein (C/EBP) or inhibitors such as the adult enhancer factor-1 (AEF-1) [34]. It is conceivable that the same set of factors is also operational in *Musca* to confer tissue-specificity based on the studies demonstrating that the expression of a reporter gene driven by the Musca *yp1* promoter is specifically confined to fat body cells in Drosophila [32].

Altogether, it appears that the relative contribution of nonautonomous and autonomous signals depends on the mode of egg production. In Drosophila where egg production does not occur in a cyclic fashion but is rather continuous, synchronization factors play a marginal role in regulating YP synthesis (Figure 5). In this system, hormonal control is mainly used to attune YP synthesis to environmental conditions such as food availability, while a much larger contribution comes from autonomously acting transcription factors to maintain a continuous supply of YP. We propose that the yp genes were initially controlled by ecdysteroids produced by the ovaries to coordinate the maturation of the oocytes with YP production. Later, *dsx* was recruited as an additional factor to control the responsiveness to ecdysteroids in male and female fat bodies. In species like Drosophila with continuous YP production, *dsx* became then the major controlling agent. In Musca, where YP production is cyclic, ecdysteroids kept their role as the main regulator.

## Figures and Tables

**Table 1:** Ecdysteroid concentration in *M* ^I^ and *F* ^man^ males. Ecdysteroid levels in hemolymph samples prepared from adult flies were measured by radioimmuno assay (see, Section 2): (**n**) number of adult flies pooled.

	Ecdysteroid concentration (pg/*μ*L)			
Fly strain	females	(*n*)	males	(*n*)
Standard XX/XY	28.5	(98)	7.5	(200)
*M* ^I^	18.8	(150)	5.1	(150)
*F* ^man^	16.2	(118)	8.2	(258)

**Table 2:** Expression of YP in a milieu with low ecdysteroids levels depends on the presence of *Md-dsx^F^* products: (**n**) number of adult flies tested for YP prod.

Genotype	Splice products of *Md-dsx *	Percentage of yolk producers	(*n*)
*M* ^I^/+ males	*Md-dsx^F^*/*Md-dsx^M^*	89.4%	47
*M* ^I^/+ males + *Md-tra2* RNAi	*Md-dsx^M^*	3.4%	59
XY males	*Md-dsx^M^*	0%	53
XY males + p[hs82::*Md-dsx^F^*]	*Md-dsx^F^*/*Md-dsx^M^*	9.8%	162^a^

^a^data compiled from 4 independent lines (see, Hediger et al. [15]).
